# Surgical Outcomes in Octogenarians with Heart Failure and Reduced Ejection Fraction following Isolated Coronary Artery Bypass Grafting—A Propensity Score Matched Analysis

**DOI:** 10.3390/jcm13164603

**Published:** 2024-08-06

**Authors:** Christian Jörg Rustenbach, Rodrigo Sandoval Boburg, Medhat Radwan, Helene Haeberle, Christophe Charotte, Ilija Djordjevic, Stefanie Wendt, Tulio Caldonazo, Ibrahim Saqer, Shekhar Saha, Philipp Schnackenburg, Lina Maria Serna-Higuita, Torsten Doenst, Christian Hagl, Thorsten Wahlers, Christian Schlensak, Stefan Reichert

**Affiliations:** 1 Department of Thoracic and Cardiovascular Surgery, German Cardiac Competence Center, Eberhard-Karls-University of Tuebingen, 72074 Tübingen, Germany; rodrigo.sandoval-boburg@med.uni-tuebingen.de (R.S.B.); medhat.radwan@med.uni-tuebingen.de (M.R.); christian.schlensak@med.uni-tuebingen.de (C.S.); stefan.reichert@med.uni-tuebingen.de (S.R.); 2 Department of Anesthesiology and Intensive Care Medicine, Eberhard-Karls-University of Tuebingen, 72074 Tübingen, Germany; helene.haeberle@med.uni-tuebingen.de (H.H.); christophe.charotte@med.uni-tuebingen.de (C.C.); 3 Department of Cardiothoracic Surgery, Heart Center, University of Cologne, 50923 Köln, Germany; ilija.djordjevic@uk-koeln.de (I.D.); stefanie.wendt@uk-koeln.de (S.W.); thorsten.wahlers@uk-koeln.de (T.W.); 4 Department of Cardiothoracic Surgery, Friedrich-Schiller-University, University Hospital of Jena, 07743 Jena, Germany; tulio.caldonazo@med.uni-jena.de (T.C.); ibrahim.saqer@med.uni-jena.de (I.S.); torsten.doenst@med.uni-jena.de (T.D.); 5 Department of Cardiac Surgery, Ludwig-Maximilians-University, 80539 München, Germany; shekhar.saha@med.uni-muenchen.de (S.S.); philipp.schnackenburg@med.uni-muenchen.de (P.S.); christian.hagl@med.uni-muenchen.de (C.H.); 6 German Centre for Cardiovascular Research (DZHK), Partner Site Munich Heart Alliance, 81377 Munich, Germany; 7 Institute for Clinical Epidemiology and Applied Biostatistics, Eberhard-Karls-University of Tuebingen, 72074 Tübingen, Germany; lina.serna-higuita@med.uni-tuebingen.de

**Keywords:** octogenarians, heart failure, CABG, HFrEF, MACCE

## Abstract

**Background/Objectives:** The demographic shift towards an aging population necessitates a reevaluation of surgical interventions like coronary artery bypass grafting (CABG) in octogenarians. This study aims to elucidate the outcomes of CABG in octogenarians with heart failure and reduced ejection fraction (HFrEF), a group traditionally considered at high risk for such procedures. **Methods:** Conducted across four academic hospitals in Germany from 2017 to 2023, this retrospective multicenter study assessed 100 patients (50 octogenarians ≥80 years and 50 non-octogenarians <80 years) with HFrEF undergoing isolated CABG. Through propensity score matching, the study aimed to compare the incidence of major adverse cardiac and cerebrovascular events (MACCEs), as well as other clinical endpoints, between the two groups. Statistical analyses included chi-square, ANOVA, Mann–Whitney U test, Cox regression, and logistic regression, aiming to identify significant differences in outcomes. **Results:** The study revealed no significant difference in the combined incidence of MACCEs between octogenarians and non-octogenarians (OR: 0.790, 95% CI: 0.174–3.576, *p* = 0.759). Mortality rates were similar across groups (7% each, *p* = 1.000), as were occurrences of postoperative myocardial infarction (2% each, *p* = 1.000) and stroke (3% total). Secondary outcomes like delirium (17% total, no significant age group difference, *p* = 0.755), acute kidney injury (18% total, *p* = 0.664), and the need for dialysis (14% total, *p* = 1.000) also showed no differences between age groups. Interestingly, non-octogenarians required more packed red blood cells during their stay (*p* = 0.008), while other postoperative care metrics, such as hospital and ICU length of stay and ventilation hours, were comparable across groups. **Conclusion:** This multicenter study highlights that CABG is a viable and safe surgical option for octogenarians with HFrEF, challenging prior assumptions about the elevated risks associated with performing this procedure in older patients. The absence of significant differences in the incidence of MACCEs and other postoperative complications across age groups emphasizes the importance of careful patient selection and perioperative management. These findings advocate for a more inclusive approach to surgical treatment for octogenarians with HFrEF, suggesting that age alone should not be a determinant for CABG eligibility. This study contributes critical insights into optimizing care for a high-risk demographic, indicating a need for tailored guidelines that accommodate the aging population with complex cardiac conditions.

## 1. Introduction

The advent of sophisticated cardiac surgical techniques and enhanced perioperative care has progressively extended the boundaries of coronary artery bypass grafting (CABG) to include a demographic once considered at prohibitively high risk. The prevalence of coronary artery disease (CAD) escalates with age, positioning octogenarians at the pinnacle of risk and incidence [1]. This demographic, characterized by a high burden of comorbidities and diminished physiological reserves, confronts the cardiovascular surgeon with a nexus of complexity. Among these, octogenarians with heart failure and reduced ejection fraction represent a more particularly challenging cohort due to their increased susceptibility to both the procedural stress of surgery and the compounded risk of comorbid conditions [2]. This manuscript endeavors to illuminate the outcomes and strategic considerations pertinent to this group, leveraging a multicenter retrospective analysis underscored by propensity score matching to distill insights from clinical practice.

Coronary artery bypass grafting (CABG) surgery, a mainstay treatment for CAD, has undergone considerable evolution over the decades, extending its applicability to a broader and more complex patient demographic [3,4]. This expansion has inevitably included the Octogenarian population, a group characterized by a higher burden of comorbidities and an increased risk of perioperative complications. Despite these challenges, CABG offers a potential improvement in quality of life and survival for selected Octogenarians patients with CAD [2]. However, the decision-making process is complicated by the paucity of evidence tailored to this subgroup, as they are often underrepresented in clinical trials.

Recent literature has increasingly focused on the outcomes of CABG in Octogenarians, particularly evaluating the balance between the procedure’s risks and its benefits. Studies have shown varied results, with some suggesting comparable outcomes to younger cohorts when patients are carefully selected, while others highlight the increased perioperative risks and the need for more individualized risk assessment models [5]. Furthermore, the advent of less invasive surgical techniques and the optimization of perioperative care have contributed to a reevaluation of the role of CABG in Octogenarian patients with complex coronary disease [6].

In this context, our study aims to shed light on the short-term outcomes of isolated CABG in Octogenarian patients with heart failure and reduced ejection fraction. Through our analysis, we seek to contribute valuable insights into the safety, efficacy, and prognostic implications of CABG in this high-risk population. By evaluating a broad range of clinical endpoints, including mortality, morbidity, major adverse cardiac and cerebrovascular events (MACCEs), and hospital readmission rates, our study aims to advance clinical understanding. Furthermore, it seeks to foster a nuanced appreciation for the intricacies of managing heart failure with reduced ejection fraction in the advanced age demographic. Additionally, our study endeavors to provide a comprehensive assessment that can inform clinical decision-making and guide future research in this evolving field.

## 2. Material and Methods

### 2.1. Study Design and Patient Population

The study was conducted through a retrospective, multicenter methodology, assessing patients aged 18 and above who were diagnosed with heart failure with reduced ejection fraction (HFrEF) and underwent isolated coronary artery bypass grafting (CABG) from 2017 to 2023. This research spanned four academic hospitals in Germany, focusing on individuals with a documented left ventricular ejection fraction (LVEF) below 40%, who exhibited signs of coronary three-vessel disease and/or left main stenosis, and were ineligible for cardiac reoperation or not experiencing cardiogenic shock, lacking a history of resuscitation, catecholamine support, or pre-surgery mechanical ventilation. After excluding participants with incomplete medical histories, a final sample of 574 patients was established. These subjects were then divided into “Octogenarian” or “Non-Octogenarian” groups, using a propensity score matching technique to ensure comparability. This division was critical for assessing CABG’s immediate impact on short-term postoperative outcomes through a comprehensive analysis of variables including demographic information, clinical assessments (EuroScore II, STS Score), prior health conditions, and specifics of the surgical intervention and recovery period. All surgeries were performed by senior physicians with extensive experience and expertise in bypass surgery. This ensured that the procedures were conducted with the highest standards of surgical care and proficiency.

The primary objective was to explore the frequency of major adverse cardiac and cerebrovascular events (MACCEs), such as in-hospital death, myocardial infarction, and strokes. Each participant was analyzed individually, with multiple MACCEs in a single patient counted as one event for the total population. This strategy enabled a direct comparison of MACCE rates between the Octogenarian and Non-Octogenarian groups, with a focus on the postoperative period. A series of appropriate statistical methods (see details below) were employed to evaluate the variance in the occurrence of at least one MACCE event between the two groups, offering a thorough risk evaluation of significant adverse events post-procedure.

Additionally, we investigated various secondary outcomes, including occurrences such as acute kidney injury (AKI), some necessitating dialysis, delirium episodes, the need for blood transfusions, ICU stay length, mechanical ventilation duration, overall hospitalization period, revascularization success, surgery length (“skin to skin” time), and sepsis incidents.

### 2.2. Propensity Score Estimation

For this study, propensity scores were derived to reduce bias from confounding factors when comparing outcomes across different age groups. Initial analysis of baseline demographics utilized chi-squared (χ^2^) tests for categorical data, analysis of variance (ANOVA) for continuous variables, and the Mann–Whitney U test for non-parametric comparisons. Logistic regression analysis was then applied to calculate propensity scores, considering a variety of demographic and clinical preoperative factors.

### 2.3. Matching Procedure

The matching process involved a 1:1 nearest neighbor method with a caliper width of 0.02, equating to 2% of the standard deviation for the logit-transformed propensity scores, aiming for the closest group alignment. The balance among cohorts was assessed through standardized mean differences, setting a threshold of less than 10% as the balance standard. This procedure strictly differentiated between on-pump and off-pump surgical methods.

### 2.4. Statistical Analysis

In line with previous methodologies, this research undertook a detailed statistical analysis to pinpoint significant differences in outcomes between groups. This involved comparing baseline, perioperative, and postoperative clinical characteristics using chi-square (χ^2^) tests, ANOVA, and the Mann–Whitney U test. Cox regression was utilized for hazards, and for non-parametric data, the Scheirer–Ray–Hare and the Kruskal–Wallis/Dunn-Bonferroni tests were applied. Missing data led to either exclusion of subjects or a sensitivity analysis to limit bias. The regression model, adjusted for all variables, incorporated only predictors with a *p*-value of ≤0.20 for the combined outcome without adjustment. Correlation checks were conducted to prevent collinearity among newly introduced metrics. Binary logistic regression was performed to address potential confounding factors and assess the impact of age on surgical outcomes, considering variables such as preoperative health status, intraoperative resource use, and postoperative complications. Statistical analyses were conducted using IBM SPSS^®^ software, version 28.0 (Armonk, NY, USA: IBM Corp.), with a significance level set at *p* ≤ 0.05.

### 2.5. Description of Surgical Procedures

A comprehensive explanation of the CABG surgical processes (ONCAB and OPCAB), detailing the techniques and protocols used, has been thoroughly discussed in our previous work. To gain a deeper understanding of these surgical practices, readers are directed to consult that document [7].

## 3. Results

Our retrospective, multicenter analysis scrutinized the outcomes of isolated coronary artery bypass grafting (CABG) in a cohort of 100 patients with heart failure with reduced ejection fraction (HFrEF), divided equally between Octogenarians (≥80 years) and Non-Octogenarians (<80 years) groups after stratified propensity score matching (PSM). The primary objective was to assess the impact of advanced age on short-term postoperative outcomes, particularly focusing on major adverse cardiac and cerebrovascular events (MACCEs), alongside a spectrum of other clinical endpoints.

### 3.1. Demographic and Clinical Profile

As illustrated in Table 1, the average age of our cohort was significantly higher in the Octogenarian group (82.26 ± 2.41) compared to the Non-Octogenarian (68.82 ± 6.92), as expected (*p* < 0.001). Gender distribution was balanced across both groups, with a slightly higher proportion of males in the Non-Octogenarians group (*p* = 0.218). Body mass index (BMI) and clinical measurements, including EuroScore II, STS Score, and preoperative left ventricular ejection fraction (LVEF), displayed no significant differences between groups, indicating a comparable baseline cardiac risk profile.

Preoperative health status parameters, including diabetes management, smoking history, hypertension, COPD, hyperlipidemia, preoperative apoplexy, carotid stenosis, peripheral vascular disease, renal insufficiency, cardiac history (left main disease, rhythm pre-operative, NSTEMI, STEMI, previous PCI), and type of surgery (elective, urgent, emergent, off-pump) showed no significant differences between groups, except for rhythm preoperative where sinus rhythm (SR) was significantly more common in the Non-Octogenarians group (*p* < 0.001), and STEMI occurrences were exclusively in the Octogenarians group (*p* < 0.001).

### 3.2. Primary Outcomes

**Figure 1:**
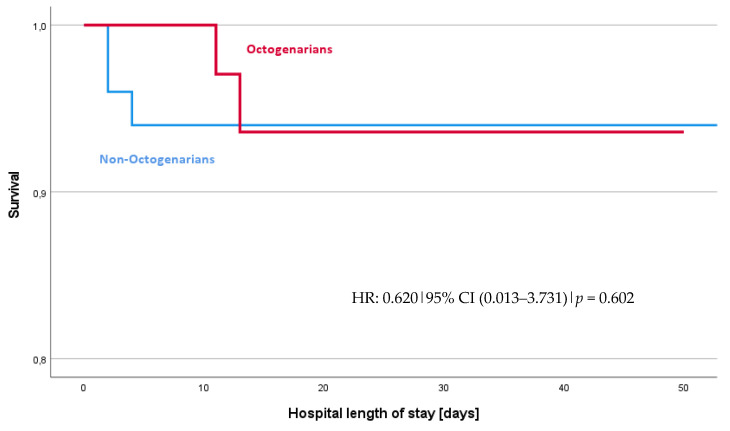
Survival curves for all-cause survival: Octogenarians vs. Non-Octogenarians. The survival analysis shows no difference between Octogenarians and Non-Octogenarians, HR = 0.620, 95% CI (0.013–3.731), *p* value = 0.602.

**Figure 2:**
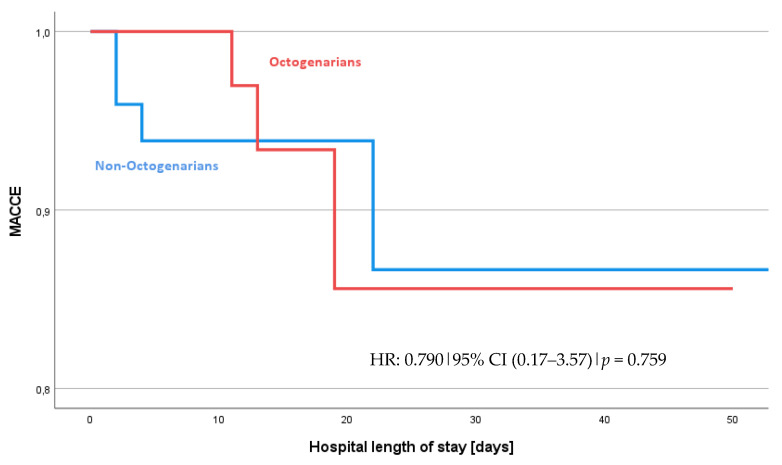
Occurrence of MACCEs: Octogenarians vs. Non-Octogenarians. The hazard ratio (HR) of 0.790 indicates the risk of experiencing MACCEs for octogenarians. They have a 21% lower risk, but there is no statistical difference.

Table 2 and Table 3, along with the odds ratios in Table 4 and data from Figure 1 and Figure 2, summarize the postoperative and surgical outcomes of our cohort. When assessing the combined incidence of MACCEs, there was no significant difference observed between the Octogenarian and Non-Octogenarian groups with an odds ratio (OR) of 0.790 and a 95% confidence interval (CI) of 0.174–3.576 (*p* = 0.759). The hazard ratio (HR) for experiencing a MACCE, as shown in Figure 2, was also not significantly different between the groups (HR: 0.790, 95% CI 0.17–3.57, *p* = 0.759). This suggests a similar risk profile for MACCEs across both age categories.

A detailed analysis of specific components of MACCEs revealed no significant difference in mortality rates between the Octogenarians and Non-Octogenarians groups, with both groups showing a 7% overall mortality rate (*p* = 1.000, Fisher’s exact test). The occurrence of postoperative myocardial infarction (MI) was equally low in both groups (2% in each, *p* = 1.000, Fisher’s exact test). Similarly, stroke incidents were not significantly different, with a 3% occurrence in the total cohort and no significant variance between the groups. The incidence rate for inotropic support was 95%, with 96% in the Octogenarians group and 94% in the Non-Octogenarians group (*p* = 0.646).

### 3.3. Secondary Outcomes

Delirium was observed in 17% of the total cohort, with no significant difference between the Octogenarians (16%) and the Non-Octogenarians (18%) groups (*p* = 0.755), indicating that age did not significantly affect the incidence of delirium postoperatively. Resuscitation post-surgery was rare and not significantly influenced by age; 3% of patients, exclusively in the Non-Octogenarians group, with no statistical significance, *p* = 0.242.

Resternotomy due to bleeding was encountered in 6% of the cohort, with a slightly higher but not statistically significant incidence in the Non-Octogenarians (8%) compared to the Octogenarians (4%) group, *p* = 0.678. This indicates that the risk of resternotomy for bleeding is similarly low across age groups.

Acute kidney injury (AKI) affected 18% of the cohort uniformly across both the Octogenarians and Non-Octogenarians groups (*p* = 0.664), demonstrating that AKI post-surgery occurred at a comparable rate regardless of age. The requirement for dialysis was also consistent across groups, with 14% in the total cohort and no significant difference between the Octogenarians and Non-Octogenarians groups (*p* = 1.000), highlighting that the need for dialysis postoperatively was evenly distributed and not influenced by age.

Sepsis was reported in 7% of patients, with a higher incidence in the Octogenarians group (10%) versus Non-Octogenarians (4%), though this difference was not statistically significant (*p* = 0.436), suggesting that the occurrence of sepsis post-surgery did not significantly differ with age.

For the entire duration of their clinical stay, postoperative parameters such as hospital length of stay (LOS), ICU LOS, ventilation hours, and time from incision to closure showed no significant differences between the Octogenarians and Non-Octogenarians groups. Specifically, hospital LOS had a median of 16 days (9–20) for the total cohort with no significant difference between the groups (*p* = 0.386, Mann–Whitney U), ICU LOS was 140 h (38–143) across the cohort with no significant age group difference (*p* = 0.422), and ventilation hours post-surgery were comparable across age groups (*p* = 0.624). The time from incision to closure was 194 min (156–248) for the total cohort, with no significant variance between the Octogenarians and Non-Octogenarians (*p* = 0.298), indicating that surgical and recovery times were consistent across age groups.

Throughout their clinical stay, the study found no significant differences between Octogenarians and Non-Octogenarians in postoperative parameters such as hospital length of stay (LOS), ICU LOS, ventilation hours, and time from incision to closure. Specifically, the median hospital LOS for the entire cohort was 16 days (ranging from 9 to 20 days) with no significant difference between the groups (*p* = 0.386, Mann–Whitney U test). The ICU LOS averaged 140 h (ranging from 38 to 143 h) across the cohort without significant differences between age groups (*p* = 0.422). Ventilation hours post-surgery were also comparable across age groups (*p* = 0.624). Furthermore, the time from incision to closure averaged 194 min (ranging from 156 to 248 min) for the total cohort, with no significant variance observed between Octogenarians and Non-Octogenarians (*p* = 0.298), indicating consistent surgical and recovery times across age groups.

Transfusion requirements during the entire clinical stay showed a significant difference only in the requirement for packed red blood cells, with the Non-Octogenarians group requiring more (710 ± 172.43 mL) compared to the Octogenarians group (396 ± 154.05 mL), *p* = 0.008, signifying a higher transfusion need in the Non-Octogenarians. Other transfusion requirements, including for pooled thrombocytes and fresh–frozen plasma, did not significantly differ, underscoring that, except for packed red blood cells, transfusion needs post-surgery were similar across age groups.

## 4. Discussion

The study’s primary finding that the incidence of major adverse cardiac and cerebrovascular events (MACCEs) does not significantly differ between Octogenarians and Non-Octogenarians is pivotal. This suggests that age, specifically being an octogenarian, does not inherently elevate the risk of MACCEs post-CABG, challenging previous assumptions about the risks associated with performing CABG in older patients. Also, no significant difference in mortality rates between the two age groups (7% in both groups) indicates that short-term postoperative mortality risk is comparable. Additionally, the occurrence of postoperative myocardial infarction (MI) and stroke incidents showed no significant variance, reinforcing the primary outcome’s implications.

Recent literature presents discrepancies regarding early postoperative outcomes. Analysis of these reveals that being in one’s 80s is independently correlated with a higher rate of MACCEs [8] and less favorable results [2,8,9], indicating that octogenarians possess a reduced physiological reserve and a higher incidence of comorbidities compared to younger individuals. Studies comparing on-pump and off-pump CABG have shown no detriment herein to being over 80 years of age in terms of short-term and early follow-up [10,11], as other studies discuss the impact of graft strategies in octogenarians showing a better outcome [12].

Despite older patients traditionally being considered at higher risk due to a greater burden of comorbidities [13], this study’s outcomes suggest that with appropriate patient selection and perioperative management, HFrEF and age should not be disqualifying factors for CABG.

The prevalence of delirium did not exhibit any substantial discrepancy between individuals in their 80s and those younger. According to the literature, the incidence of postoperative delirium (POD) is high [14] and can reach up to 33.5% following cardiac surgery [15]. Our analysis shows a lower incidence rate of 18%, acknowledging that POD becomes more common with advancing age in cardiac surgery and is associated with significantly worse outcomes, as demonstrated by large meta-analyses [16,17].

The rates of acute kidney injury (AKI), the need for dialysis, and sepsis incidents were similar across age groups, emphasizing the ability to manage these complications effectively in octogenarians as in younger patients. AKI was common in elderly patients [18], and several investigations demonstrate an independent effect of impaired renal function on mortality in patients with CAD and heart failure (HF), demonstrating it as a risk factor for mortality [19]. In contrast to previous studies that reported discrepancies with significantly higher mortality, more instances of acute kidney injury (AKI), and markedly more neurological complications among older populations [20], this study demonstrates no significant differences in these postoperative outcomes as it further supports the notion that age does not significantly affect the risk of these specific postoperative complications in this special cohort.

Although non-octogenarians required more packed red blood cells during their clinical stay, other transfusion needs and postoperative care metrics such as hospital and ICU length of stay, and ventilation hours were comparable. This aligns with other studies, investigating the survival and length of stay in octogenarians following blood transfusion [21].

The similarity in times from incision to closure and the percentage of complete revascularization even in on- or off-pump strategy between groups indicate that the surgical process and recovery potential are consistent across age demographics, supporting the technical feasibility of CABG in octogenarians.

Octogenarians undergoing emergent or urgent CABG face significantly higher risks due to acute conditions, multiple comorbidities, and reduced physiological reserve. Emergent surgeries in octogenarians present technical difficulties like hemodynamic instability and poor tissue quality, leading to prolonged operative times and increased risk. Extracorporeal life support (ECLS) is crucial for stabilizing high-risk patients during and after surgery, improving hemodynamic stability and outcomes despite increased care complexity despite there being no difference in elective, urgent, or emergent patients in this study. Multidisciplinary teams must carefully select and assess octogenarian patients for emergent CABG, considering all health aspects to optimize postoperative recovery. Our findings indicate higher morbidity and mortality in emergent cases but justify surgery in selected patients through significant clinical improvement. Our study supports CABG as a viable, safe option for well-selected octogenarian patients with HFrEF, advocating for guideline updates to include these patients based on comprehensive assessments beyond age alone.

The findings from this study could significantly influence clinical decision-making processes by providing evidence that CABG is a viable and safe option for selected octogenarian patients with HFrEF. The lack of significant differences in the incidence of MACCE and other postoperative complications between octogenarians and younger patients supports a more inclusive approach toward offering CABG to older patients. These insights could potentially inform updates to guidelines on CABG in elderly populations, advocating for a nuanced approach to patient selection that considers more than just chronological age. This could lead to an expansion of CABG eligibility criteria to include well-selected octogenarian patients, thereby enhancing their access to potentially life-improving surgery.

## 5. Future Directions and Limitations

The evolving demographic trends toward an older population necessitate further research focused on the long-term outcomes of CABG in octogenarians with HFrEF. Investigating the durability of surgical benefits, the impact of emerging less invasive surgical techniques, and the optimization of perioperative management protocols will be vital in refining treatment strategies for this high-risk group. Furthermore, the development and validation of predictive models for patient selection and risk stratification will enhance clinical decision-making, aiming to maximize the benefits while minimizing the risks of CABG in elderly patients with complex coronary disease.

Despite the strengths of utilizing a multicenter design and propensity score matching to minimize confounding biases, our study’s retrospective nature and the inherent limitations of observational research may not capture all potential variables affecting outcomes. The relatively modest sample size and the short-term focus of the study limit the generalizability of our findings and the ability to draw conclusions about long-term outcomes post-CABG in octogenarians. The diversity in surgical techniques and perioperative care across participating centers may also introduce variability that could influence the observed outcomes.

## 6. Conclusions

Our multicenter retrospective analysis provides a comprehensive examination of isolated coronary artery bypass grafting (CABG) outcomes in octogenarians with heart failure and reduced ejection fraction (HFrEF), a demographic traditionally considered at high risk for surgical interventions. The study’s findings reveal no significant differences in the incidence of major adverse cardiac and cerebrovascular events (MACCEs), mortality, or other postoperative complications between octogenarians and a younger cohort. This suggests that CABG can be safely and effectively performed in carefully selected octogenarian patients, offering them benefits comparable to those observed in younger patients. These results underscore the feasibility of CABG as a therapeutic option for enhancing the quality of life among octogenarians with complex coronary disease and HFrEF, provided that a meticulous patient selection process and optimized perioperative care are in place.

Based on our findings, bypass surgery emerges as a robust and viable treatment option, demonstrating safety and efficacy that warrant its active consideration even in octogenarian patients with HFrEF. This paradigm shift encourages clinicians to offer CABG to well-selected elderly patients, potentially transforming their clinical outcomes and quality of life.

## Figures and Tables

**Table 1:** Preoperative demographic data and clinical measurements.

Variable n (%)|Mean (±SD)	Total Cohort	Octogenarians	Non-Octogenarians	*p* Value
	(n = 100)	(n = 50)	(n = 50)	
*Demographic Data*				
Age	75.54 (±8.49)	82.26 (±2.41)	68.82 (±6.92)	<0.001 ^ANOVA^
Gender				
Male	88 (88%)	42 (84%)	46 (92%)	0.218 ^Chi2^
Female	12 (12%)	8 (16%)	4 (8%)	
BMI	27.16 (±3.54)	27.17 (±3.51)	27.15 (±3.59)	0.997 ^ANOVA^
*Clinical Measurements*				
EuroScore II	8.27 (±6.90)	9.36 (±7.13)	7.17 (±6.55)	0.114 ^ANOVA^
STS Score	3.62 (±3.04)	3.93 (±3.02)	3.30 (±3.06)	0.299 ^ANOVA^
LVEF preop	29.66 (±7.10)	30.44 (±7.44)	28.88 (±6.73)	0.274 ^ANOVA^
*NYHA Class*				
III	45 (45%)	24 (48%)	21 (42%)	0.868 ^Chi2^
IV	13 (13%)	7 (14%)	6 (12%)	
*Health Status*				
Diabetes				
OAD	28 (28%)	16 (32%)	12 (24%)	0.654 ^Chi2^
Insulin-dependent	20 (20%)	9 (18%)	11 (22%)	
Smoking history				
Non	69 (69%)	36 (72%)	33 (66%)	0.515 ^Chi2^
Former	23 (23%)	9 (18%)	14 (28%)	
Active	5 (5%)	2 (4%)	3 (6%)	
Hypertension	94 (94%)	47 (94%)	47 (94%)	1.000 ^Chi2^
COPD	12 (12%)	6 (12%)	6 (12%)	1.000 ^Chi2^
Hyperlipidemia	80 (80%)	39 (79.6%)	41 (82%)	0.761 ^Chi2^
Apoplexy, preoperative	13 (13%)	8 (16%)	5 (10%)	0.372 ^Chi2^
Carotid stenosis	22 (22%)	10 (20%)	12 (24%)	0.629 ^Chi2^
Peripheral vascular disease	23 (23%)	10 (20%)	13 (26%)	0.476 ^Chi2^
Renal insufficiency	37 (37.8%)	18 (37.5%)	19 (38%)	0.959 ^Chi2^
*Cardiac History*				
Left main disease	41 (41%)	24 (48%)	17 (34%)	0.155 ^Chi2^
Rhythm, preoperative				
SR	62 (62%)	20 (40.8%)	42 (84%)	<0.001 ^Chi2^
NSTEMI	40 (40%)	22 (44%)	18 (36%)	0.414 ^Chi2^
STEMI	11 (11%)	11 (22%)	0 (0%)	<0.001 ^Chi2^
Previous PCI	34 (34%)	18 (36%)	16 (32%)	0.673 ^Chi2^
*Type of Surgery*				
Elective	57 (57%)	29 (58%)	28 (56%)	0.552 ^Chi2^
Urgent	28 (28%)	12 (24%)	16 (32%)	
Emergent	15 (15%)	9 (18%)	6 (12%)	
Off-pump	62 (62%)	27 (54%)	35 (70%)	0.100 ^Chi2^

**Table 2:** Intra- and postoperative outcomes.

Variable	Total Cohort	Octogenarians	Non-Octogenarians	*p* Value
	(n = 100)	(n = 50)	(n = 50)	
*Intraoperative Requirement for Transfusion [mL], mean (±SD)*				
Packed red blood cells	90 (±215.32)	96 (±222.19)	84 (±210.31)	0.782 ^ANOVA^
Pooled thrombocytes	63 (±166.76)	90 (±203.29)	36 (±115.63)	0.091 ^ANOVA^
Fresh–frozen plasma	63 (±296.32)	90 (±333.35)	46 (±254.55)	0.365 ^ANOVA^
*Postoperative (after chest closure) Vasopressor and Inotropic requirements [γ], median (IQR)*				
Epinephrine	0.11 (0–0.22)	0.06 (0–0.20)	0.05 (0–0.15)	0.585 ^MW^
Norepinephrine	0.10 (0–0.20)	0.12 (0–0.24)	0.1 (0–0.2)	0.318 ^MW^
*Postoperative Parameters, mean (IQR)*				
Hospital LOS (d)	16 (9–20)	14 (10–19)	11.5 (8–23)	0.386 ^MW^
ICU LOS (h)	140 (38–143)	69 (27–138)	76 (42–150)	0.422 ^MW^
Vent (h)	42 (6–25)	11.5 (7.75–19)	15 (4.75–38)	0.624 ^MW^
Incision to closure (min)	194 (156–248)	201 (166–250)	188 (139–248)	0.298 ^MW^
Complete revascularization, n (%)	80 (80%)	41 (82%)	39 (78%)	0.617 ^Chi2^
Number of bypass grafts	2.63 (±0.894)	2.76 (±0.916)	2.52 (±0.863)	0.691 ^TT^
*Transfusion Requirements during the Entire Clinical Stay [mL], mean (±SD)*				
Packed red blood cells	553 (±191.70)	396 (±154.05)	710 (±172.43)	0.008 ^ANOVA^
Pooled thrombocytes	77.50 (±206.26)	100 (±242.22)	55 (±162.02)	0.278 ^ANOVA^
Fresh–frozen plasma	92.50 (±195.75)	95 (±102.58)	90 (±136.34)	0.960 ^ANOVA^

**Table 3:** Postoperative complications.

Variable n (%)	Total Cohort	Octogenarians	Non-Octogenarians	*p* Value
	(n = 100)	(n = 50)	(n = 50)	
Resuscitation	3 (3%)	0 (0%)	3 (6%)	0.242 ^Fish^
Resternotomy due to bleeding	6 (6%)	2 (4%)	4 (8%)	0.678 ^Fish^
ECLS	4 (4%)	2 (4%)	2 (4%)	1.000 ^Fish^
Elective	2	1	1	0.552 ^Chi2^
Urgent	1	0	1	
Emergent	1	1	0	
AKI	18 (18%)	9 (18%)	9 (18%)	0.664 ^Chi2^
Dialysis	14 (14%)	7 (14%)	7 (14%)	1.000 ^Chi2^
Stroke	3 (3%)	1 (2%)	2 (4%)	1.000 ^Fish^
Delirium	17 (17%)	8 (16%)	9 (18%)	0.755 ^Chi2^
Sepsis	7(7%)	5 (10%)	2 (4%)	0.436 ^Fish^
Postoperative MI	2 (2%)	1 (2%)	1 (2%)	1.000 ^Fish^
Mortality	7 (7%)	4 (8%)	3 (6%)	1.000 ^Fish^
MACCE	11 (11%)	5 (10%)	6 (12%)	0.749 ^Chi2^
Incidence for inotropic support	95 (95%)	48 (96%)	47 (94%)	0.646 ^Chi2^

**Table 4:** Odds and 95% CI of complications categorized by Octogenarians vs. Non-Octogenarians.

Variable	OR	95% CI	B	*p* Value
Resuscitation	1.411	0.059–2.81	1.618	0.391
Resternotomy	1.570	0.072–34.44	0.451	0.775
ECLS	0.628	0.037–10.767	−0.466	0.748
AKI	0.885	0.060–9.010	−0.123	0.918
Dialysis	0.789	0.056–14.494	−0.101	0.043
Stroke	0.170	0.051–8.661	−1.772	0.295
Delirium	2.129	0.446–10.166	0.756	0.144
Sepsis	3.705	0.583–61.57	2.273	0.103
Postoperative MI	1.317	0.942–9.425	1.015	0.791
Mortality	1.019	0.121–10.681	−1.310	0.614
MACCE	0.790	0.174–3.576	−0.236	0.759
Hospital LOS	1.013	0.966–1.063	0.013	0.590
ICU LOS	0.999	0.995–1.003	−0.001	0.636
Vent.	0.999	0.987–1.005	−0.004	0.360
Inotropic support	0.653	0.104–4.082	0.425	0.648

## Data Availability

Data are contained within the article, and the foundational research data can be made available upon request in compliance with the EU’s General Data Protection Regulation (GDPR). To ensure compliance, we will seek legal counsel in this matter.
